# Self-delivering RNAi immunotherapeutic PH-762 silences PD-1 to generate local and abscopal antitumor efficacy

**DOI:** 10.3389/fimmu.2024.1501679

**Published:** 2024-12-04

**Authors:** Benjamin Cuiffo, Melissa Maxwell, Dingxue Yan, Ramdane Guemiri, Andrew Boone, Deborah Bellet, Brianna Rivest, James Cardia, Caroline Robert, Simon P. Fricker

**Affiliations:** ^1^ Phio Pharmaceuticals, Marlborough, MA, United States; ^2^ Dermatology Unit, Gustave Roussy Cancer Center, Villejuif, France

**Keywords:** PD-1 inhibition, siRNA, abscopal effect, immunooncology, immunotherapy

## Abstract

**Objective:**

Immunotherapeutic inhibition of PD-1 by systemically administered monoclonal antibodies is widely used in cancer treatment, but it may cause severe immune-related adverse events (irSAEs). Neoadjuvant PD-1 inhibition before surgery has shown promise in reducing recurrence by stimulating durable antitumor immunity. Local intratumoral (IT) immunotherapy is a potential strategy to minimize irSAEs, but antibodies have limited tumor penetration, making them less suitable for this approach. Therapeutic self-delivering RNAi (INTASYL) is an emerging modality well-suited for neoadjuvant immunotherapy. This study presents preclinical proof-of-concept for PH-762, an INTASYL designed to silence PD-1, currently in clinical development for advanced cutaneous malignancies (ClinicalTrials.gov#NCT06014086).

**Methods and analysis:**

PH-762 pharmacology was characterized *in vitro*, and *in vivo* antitumor efficacy was evaluated using a murine analogue (mPH-762) in syngeneic tumor models with varying PD-1 responsiveness. Bilateral Hepa1-6 models assessed abscopal effects of local treatment. Ex vivo analyses explored mechanisms of direct and abscopal efficacy.

**Results:**

PH-762 was rapidly internalized by human T cells, silencing PD-1 mRNA and decreasing PD-1 surface protein, enhancing TCR-stimulated IFN-γ and CXCL10 secretion. *In vivo*, IT mPH-762 provided robust antitumor efficacy, local and lymphatic biodistribution, and was well tolerated. Ex vivo analyses revealed that IT mPH-762 depleted PD-1 protein, promoted leukocyte and T cell infiltration, and correlated with tumor control. IT mPH-762 also demonstrated efficacy against untreated distal tumors (abscopal effect) by priming systemic antitumor immunity.

**Conclusion:**

These data support PH-762 as a promising candidate for neoadjuvant immunotherapy in clinical studies.

## Introduction

Immune checkpoint inhibition (ICI) with monoclonal antibodies such as those directed at PD-1 has revolutionized the treatment and outcomes of some cancers. By blocking inhibitory receptors expressed by anti-tumor T cells, these antibodies aim to break the immune tolerance of tumor cells and allow the generation of durable cancer immunity. However, systemic immunotherapies often elicit severe immune related adverse events (irSAEs) (1, 2), which may force discontinuation in affected patients and limit benefit for patients at high risk for irSAEs, such as those with pre-existing auto-immune diseases (3).

For patients with resectable solid tumors, ICI provided as adjuvant therapy improves post-surgical outcomes and may decrease recurrence (4). However, the benefit of systemically administered adjuvant ICI may also be limited by treatment emergent irSAEs (5–9). Neoadjuvant ICI has shown additional promise as a strategy to mitigate tumor recurrence and improve outcomes for patients with resectable solid tumors (10, 11). Neoadjuvant immunotherapy is an attractive treatment paradigm as it carries the potential to prime antitumor immunity in the presence of tumor neoantigens, and thereby elicit durable anti-tumor immunity (12). Additionally, preoperative neoadjuvant therapy may shrink the tumor and result in reduced surgical margins (13). However, irSAEs are also limiting in the neoadjuvant setting, especially in studies combining ipilimumab + nivolumab, where between 73% and 90% grade 3/4 adverse events are observed (14, 15), representing a risk factor that may result in the need to delay surgery. Additional therapeutic options are required to mitigate systemic irSAEs resulting from ICI in the neoadjuvant setting.

Local intratumoral (IT) immunotherapy provides a strategy to circumvent irSAEs mediated by systemic immunotherapy (16, 17). However, large molecular weight antibodies show limited local tumor penetration and appear ill-suited for this application (18, 19). Therapeutic self-delivering RNAi (INTASYL™ Phio Pharmaceuticals) is an emerging modality that has been previously been demonstrated to provide efficient delivery into target cells without need for specialized formulations or drug delivery systems in both preclinical and clinical studies; (ClinicalTrial#NCT02246465). PH-762 comprises a combination of stabilizing modifications, cholesterol, and a single-stranded phosphorothioate tail (INTASYL™ platform; Phio Pharmaceuticals) enabling “self-delivery” through cellular internalization via endocytosis. We show that these modifications enable rapid (within minutes) and efficient (100% of cells) uptake by T cells without the need for specialized formulation or delivery vehicles. INTASYL compounds including PH-762 are formulated in PBS.

Here we describe preclinical proof-of-concept studies supporting the clinical development of PH-762, an INTASYL compound designed to silence human PD-1 with high specificity. PH-762 is rapidly internalized by endocytosis and exerts its PD-1 gene-silencing function via the endogenous small interfering RNA (siRNA) gene regulation pathway (20). The high target-sequence specificity of PH-762 is supported by in silico genome-wide analyses of binding free energies of possible off-targets of both the guide and passenger strands of the PH-762 duplex, including both coding and noncoding transcripts, with the most probable off-target (GRAMD4) predicted to interact with PH-762 with 1000x weaker binding affinity compared with its target PDCD1 mRNA. Experimentally, an IC50 could not be determined for PH-762 activity toward GRAMD4 mRNA in human pan T cells. This data indicates PH-762 carries negligible risk of sequence-mediated off-target effects. PH-762 is currently under clinical investigation as neoadjuvant immunotherapy for patients with resectable cutaneous malignancies (ClinicalTrials.gov#NCT06014086).

## Results

### PH-762 is a stabilized self-delivering siRNA compound designed to potently silence PDCD1 mRNA with high specificity

PH-762 is a chemically modified asymmetric duplex oligonucleotide, consisting of a 20 nucleotide antisense (guide) strand and a 15 nucleotide sense (passenger) strand. The compound contains a 15 base pair duplex, and a single-stranded phosphorothioate tail. In addition, chemical modifications are incorporated throughout the compound, including 2′-fluoro and 2′-O-methyl modifications (providing stabilization), and the 3′ end of the passenger strand is conjugated to cholesterol via a triethylene glycol linker. The combination of the stabilizing modifications, cholesterol and single-stranded phosphorothioate tail enable cellular internalization via endocytosis. These modifications result in rapid and efficient uptake by cells and tissues without the need for further formulation or delivery vehicles, and PH-762 is formulated only in PBS.

PH-762 was designed to precisely target human *PDCD1* (PD-1) mRNA with high affinity. The screening of compounds targeting PDCD1 and selection of PD78fm as the lead was conducted as described by Ligtenberg et al. (21) Potential sequence mediated off-target effects were evaluated by in silico analysis of binding free energies using Visual OMP and Thermoblast software. Both guide and passenger strands of the PH-762 duplex were analyzed for off-target effects across the entire human transcriptome, including off target antagonist or agonist effects toward noncoding transcripts (such as miRNA or lncRNA). No noncoding off target effects were detected. *GRAMD4* was identified as a weak possible off-target, however, PH-762 was predicted to interact with *GRAMD4* mRNA with a 1000x weaker binding affinity compared to its *PDCD1* mRNA target. The in silico predictions were confirmed experimentally in CD3/CD28 bead-activated human pan T cells, where no off-target silencing *GRAMD4* mRNA was observed, such that an IC_50_ for PH-762 toward *GRAMD4* mRNA could not be determined (**Supplementary Figure S1**). These data suggest that PH-762 does not carry significant sequence-mediated off-target effects.

#### PH-762 is rapidly and efficiently internalized by human pan T cells

The cell internalization kinetics of PH-762 were assessed in PD-1-expressing human pan T cells. A fluorescently labeled PH-762 compound (fl-PH-762; labeled on the passenger strand) was applied to CD3/CD28 bead-activated human pan T cells; uptake of fl-PH-762 was compared to that of identically labeled standard unmodified (canonical) siRNA.

Maximal internalization of fl-PH-762 was achieved ~5 minutes post-treatment; in contrast, even at 24 hours, canonical RNAi did not achieve maximal internalization, showing a ~22-fold reduced level of signal compared to fl-PH-762 (**Supplementary Figures S2A, B**). Analysis by flow cytometry indicated uptake of fl-PH-762 by 100% of the cells (**Supplementary Figure S2C**). These data show that PH-762 is rapidly and efficiently internalized by activated T cells.

#### PH-762 silences PD-1 mRNA and surface protein in activated human pan T cells *in vitro*

PH-762-mediated silencing of PD-1 mRNA and surface protein was assessed in CD3/CD28-bead activated human pan T cells derived from the peripheral blood of three individual donors. A chemically matched non-targeting control (NTC) INTASYL was applied to the cells as a control condition to identify any target-sequence independent effects.

PH-762 treatment (72 h) conferred concentration-dependent silencing of PD-1 mRNA of ~80% at 2 μM, the highest concentration tested, compared to PBS-treated controls (UTC). IC_50_ of PD-1 mRNA silencing by PH-762 in CD3/CD28-activated human pan T cells was 0.1829-0.5452 μM across donors (representative data from single donor shown in **Supplementary Figure S2D**). PH-762 treatment similarly conferred a concentration-dependent reduction of PD-1 surface protein up to ~48% at 2 μM when administered to activated human pan T cells. IC_50_ of PD-1 surface protein reduction by PH-762 was 2.080 – 2.433 μM across donors (representative data from single donor shown in **Supplementary Figure S2E**).

These data show robust concentration associated on-target silencing activity of PH-762 toward PDCD1 mRNA in activated human pan T cells from three individual donors, resulting in a corresponding reduction in PD-1 surface protein.

#### PH-762 enhances functional secretion of immune response cytokines IFN-γ and CXCL10 in TCR-stimulated human pan T cells

IFN-γ and CXCL10 are well characterized mediators of anti-tumor immune responses and have been identified as components of clinical signatures of response to PD-1 inhibition (22). IFN-γ is a master regulator of immune responses, and CXCL10 serves as an attractant chemokine for leukocytes including T cells and NK cells. The functional impacts of PD-1 silencing by PH-762 were assessed in human pan T cells derived from two individual donors by assaying IFN-γ and CXCL10 secretion in TCR-stimulated (plate-bound OKT3) human pan T cells simultaneously treated with PH-762 or NTC for 72 h. Unstimulated, PBS-treated cells (US) and OKT3-stimulated, PBS-treated (UTC) conditions served as controls. Activation-induced expression of PD-L1 on T cells has been described to promote self-tolerance of neighboring T cells in cancer (23). PH-762 treatment increased IFN-γ (**Supplementary Figure S2F**) and CXCL10 (**Supplementary Figure S2G**) levels by ~4-fold and ~7-fold respectively compared to UTC. PD-1 silencing was confirmed in parallel at both the mRNA and protein level (**Supplementary Figure S2**) (In contrast, PH-762 did not elicit significant release of cytokine release syndrome (CRS) associated cytokines IL-2, IL-6, IL-10, TNF-α, or IFN-γ from unstimulated human PBMCs derived from ten individual donors (**Supplementary Figure S3**) suggesting that functional effects of PH-762 are confined to activated T cells (that express PD-1), such as those that have been TCR-stimulated.

These data demonstrate that PH-762 enhances function immune responses of TCR-stimulated human pan T cells derived from multiple donors.

#### Intratumoral administration of mouse-targeted PH-762 provides antitumor efficacy in multiple syngeneic tumor models of varying responsivity to PD-1 inhibition

PH-762 was designed to silence human PD-1 mRNA with high specificity. In silico target sequence homology analyses performed using BLASTn (NCBI) predicted that PH-762 carries insufficient sequence homology to mediate silencing of mouse PD-1 ortholog mRNA. Therefore, a murine PD-1-targeted analogue of PH-762 (mPH-762) was synthesized with identical structural and chemical modifications as PH-762, but with a nucleotide sequence conferring target specificity toward murine PD-1 mRNA, to enable *in vivo* efficacy studies in tumor-bearing mouse models. The murine PD-1 silencing activity of mPH-762 was confirmed in CD3/CD28 bead activated EL4 murine T cells (**Supplementary Figure S4**).

**Figure 1:**
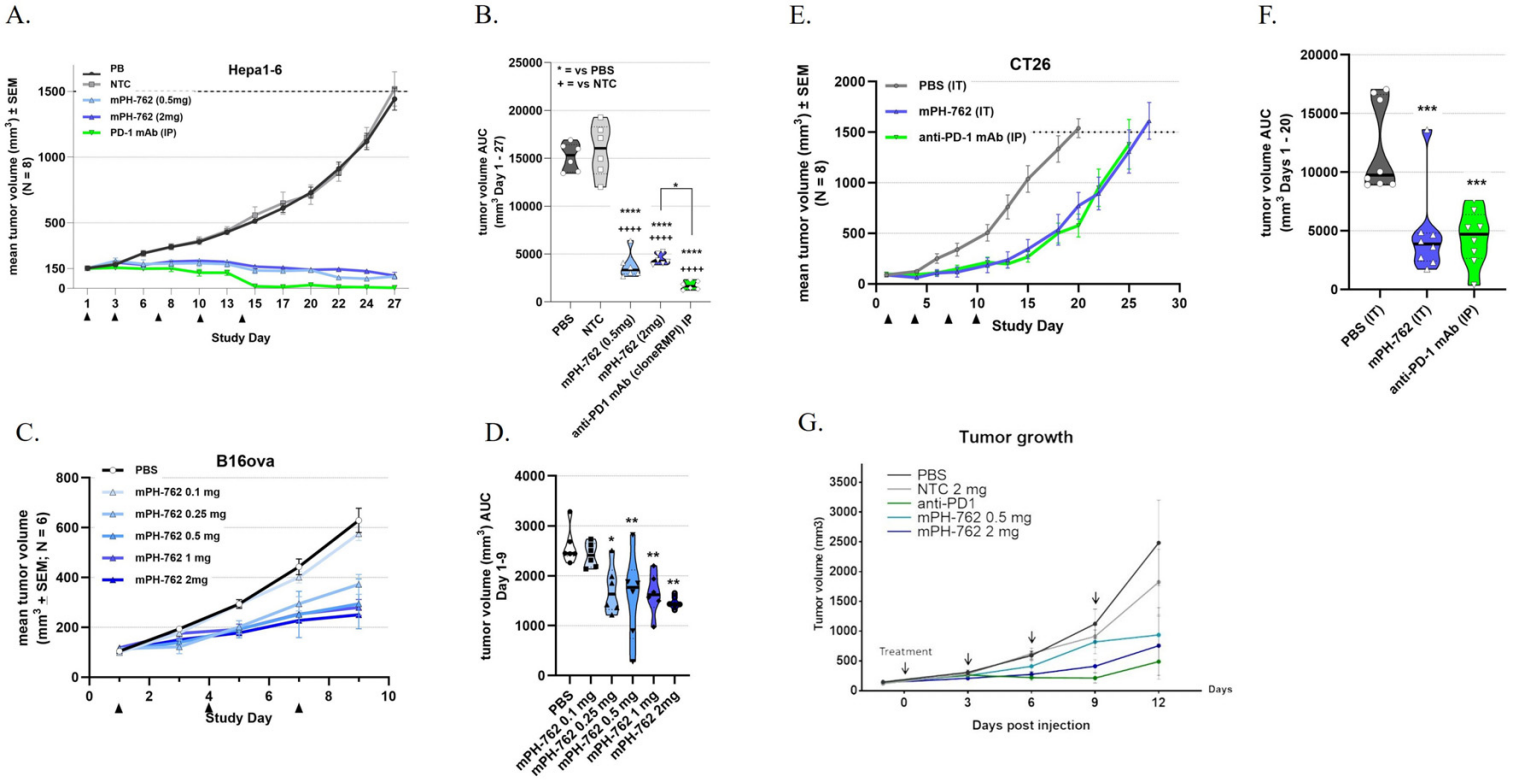
IT mPH-762 treatment elicits tumor control in a dose-associated manner in multiple syngeneic tumor models. mPH-762 was administered IT q3d as indicated. Anti-PD-1 mAb control was administered IP q3d as indicated. Tumors were measured 3x/week by caliper, and volumes calculated by V = (LxW2)/2. **(A, B)** Hepa1-6 model. **(C, D)** B16-OVA model. **(E, F)** CT26 model. **(G)** BP model **(A, C, E, G)** Group mean tumor volume ± SEM (N = as indicated) over time. **(B, D, F)** Tumor volume area under the curve (AUC) calculated for each animal by trapezoidal transformation over the indicated time period. Violin plots with individual animals and medians are shown. Statistical significance of differences in mean AUC were assessed by one-way ANOVA and Tukey’s or Dunnett’s **(F)** multiple comparisons *post-hoc* tests. ****p <0.0001; ***p <0.001; **p <0.01.

Syngeneic murine tumor models of varying responsiveness to anti-PD-1 antibody (PD-1 mAb) therapy were employed to evaluate the efficacy and tolerability of intratumoral (IT) treatment with mPH-762: Braf^V600E^/Pten^-/-^ “BP” melanoma, (PD-1 mAb-responsive melanoma) (Cooper et al., 2014), Hepa1-6 (PD-1 mAb-responsive hepatocellular carcinoma); B16-OVA (PD-1 mAb moderately responsive melanoma); and CT26 (PD-1 mAb modestly responsive colon carcinoma) were utilized. In all models, mPH-762 was well tolerated at the maximum administered dose of 2 mg/dose (0.05 mL of 40 mg/mL: q3d x5 (Hepa1-6, C57BL/6), q3d x4 (BP melanoma; C57BL/6; CT26, BALB/c) or q3d x3 B16-OVA, C57BL/6), such that treatments did not significantly impact cumulative weight gain compared to treatment with vehicle, NTC, or systemic (intraperitoneal; IP) anti-PD-1 mAb positive control (**Supplementary Figure S5**). Additionally, there was no evidence of injection site reactions found in any model treated with mPH-762. For all tumor models, IT mPH-762 significantly inhibited mean cumulative tumor volume growth compared to PBS or NTC, with response levels correlating to the relative anti-PD-1 mAb responsiveness of the model (**Figure 1**).

These data support the suitability of IT PH-762 to treat solid tumors by demonstrating robust, on-target, dose-associated antitumor efficacy in syngeneic mouse tumor models of injectable indications that are often clinically amenable to surgical resection: melanoma, hepatocellular carcinoma, and colon carcinoma.

#### Biodistribution of fluorescent IT mPH-762 in the subcutaneous BP melanoma model

The biodistribution kinetics of fluorescent reporter-conjugated murine targeted PH-762 (fl-mPH-762; reporter conjugated to the passenger strand) were assessed following single IT administration in the subcutaneous BP melanoma model.

Fl-mPH-762 could be detected within minutes following IT administration in live mice at the site of the injection by *in vivo* imaging (**Supplementary Figure S6B**) or by eye (**Supplementary Figure S6A**). After 24 h, the fluorescence had spread locoregionally and was still detectable at similar intensity even after 72 h. Diffusing fluorescence was observed in the skin around the injection spot and in lymph nodes (**Supplementary Figure S6C**). The fluorescence was also found in the urine of the mouse as early as 4 h after the injection (**Supplementary Figure S6B**), suggesting diffusion into the blood circulation.

At each time point, two (2) mice were euthanized, and lymph nodes collected from sites of varying proximity to the tumor to measure the relative fluorescence of the compound. Resected lymph nodes showed a gradient distribution of the compound in the lymphatic system, marked by the pink coloration of all the lymph nodes (locoregional and distant); relative fluorescence was inversely correlated to the distance from the tumor-regional lymph node (**Supplementary Figure S6D**). The coloration intensity decreased over time and was still observable after 72 h.

Lymph nodes were then dissociated and assessed for intracellular compound fluorescence was assessed in all cells, as well specifically in CD8^+^ or CD4^+^ T cells by flow cytometry. For all lymph node cells assessed, fluorescence was inversely correlated to distance from the tumor; the more distant the lymph node was from the tumor, the less fluorescence detected. The fluorescence also decreased over time (**Supplementary Figures S6E–G**).

These observations show tissue biodistribution of IT fl-mPH-762 diffusing in the local area of the injected tumor and through the lymphatic system, with lymph nodes proximal to the tumor showing the highest levels of fluorescent-tagged compound.

#### Intratumoral mPH-762 treatment increased T cell infiltration into the tumor microenvironment and decreased surface PD-1 levels

**Figure 2:**
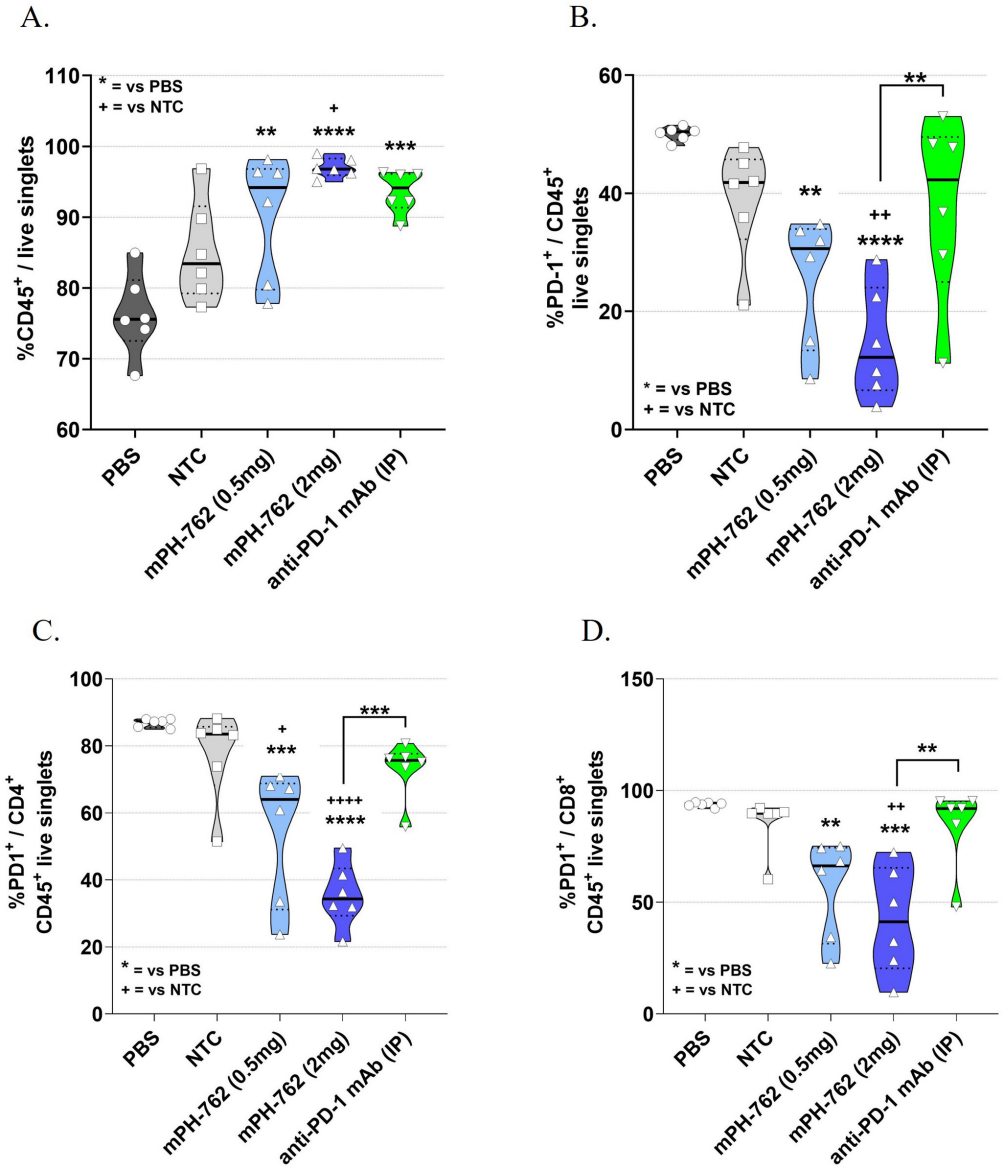
IT mPH-762 decreases PD-1 expression and increases infiltrating leukocytes in the TME. Leukocytes were enriched from Day 13 dissociated bulk tumor cells by density gradient centrifugation. Following immunostaining, flow cytometric analyses were performed to assess percentage (%) of singlet events as indicated. Box and whisker plots with medians and individual animals indicated are shown for each analysis by group. The statistical significance of differences in group means were assessed by one-way ANOVA and Tukey’s multiple comparisons *post-hoc* tests. **(A)** % CD45^+^ lymphocytes of live singlet cells. **(B)** %PD-1^+^/CD45^+^ TILs. **(C)** %PD-1^+^/CD4^+^ TILs. **(D)** %PD-1^+^/CD8^+^ (right) tumor T cells. ****p<0.0001, ***p<0.001, **p<0.01, *p<0.1.

Tumors were collected from the Hepa1-6 *in vivo* model from a satellite cohort of mice on Day 13; leukocytes were enriched by density gradient centrifugation and the leukocyte enriched tumor microenvironment (TME) characterized by immunostaining/flow cytometry (gating shown in **Supplementary Figure S7**). IT treatment with mPH-762 produced a dose-associated statistically significant increase in the mean percentage (%) CD45^+^ tumor immune lymphocyte infiltrate (TIL), analogous to that observed with the systemic treated anti-PD-1 control (**Figure 2A**). This was accompanied by a dose-associated statistically significant on-target reduction of PD-1 surface protein in tumor CD45^+^ leukocytes (**Figure 2B**), including CD4^+^ and CD8^+^ T cells (**Figures 2C, D**), for mice treated with PH-762.

These data suggest that IT mPH-762 exerts antitumor effects *in vivo* by both promoting infiltration of leukocytes, including CD8^+^ and CD4^+^ T cells, into the TME, and providing on-target reduction of TIL surface PD-1.

#### Intratumoral mPH-762 provides abscopal efficacy to untreated distal tumors in bilateral Hepa1-6 models

**Figure 3:**
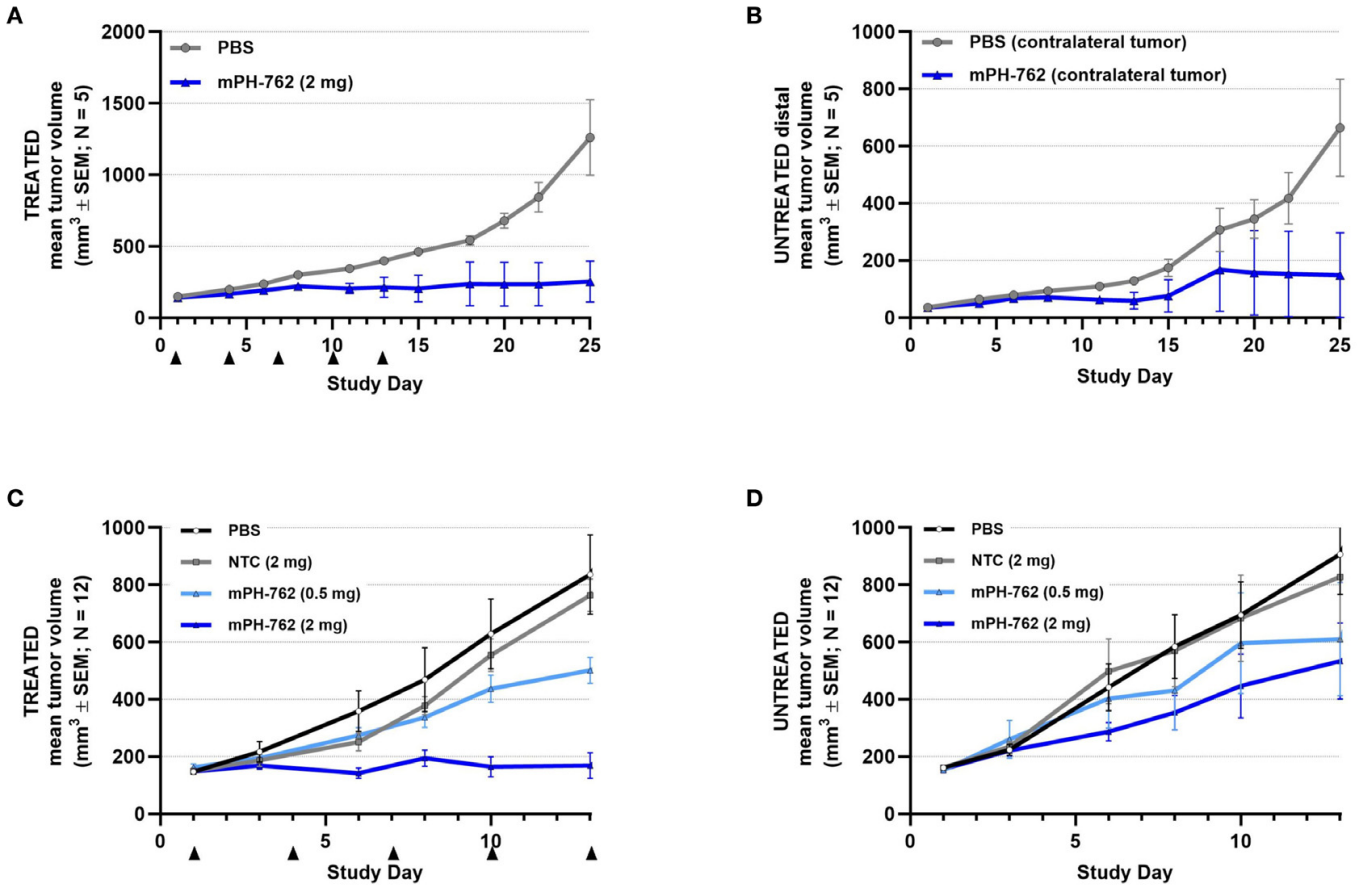
mPH-762 provides direct and abscopal efficacy in a bilateral SC Hepa1-6 model. Mice were implanted with Hepa1-6 cells subcutaneously into bilateral flanks with 1e07 (directly treated tumor; **(A)** and 1e06 (untreated distal tumor; **(B)** or 1e07 (both flanks; second study; **(C, D)**. mPH-762 or PBS vehicle were administered IT q3d x 5 to the left (treated) tumor only. Directly treated **(A, C)** and untreated distal **(B, D)** tumors were measured 3x/week, and volume (V) calculated by V = (LxW2)/2. Group mean tumor volume ± SEM is shown over time.

Two bilateral subcutaneous Hepa1-6 models were performed; tumors were implanted on opposite flanks of each mouse and mPH-762 or control treatments were administered IT to one tumor only (the directly treated tumor), while the other tumor remained untreated (the untreated distal tumor). In the first (pilot) study, the untreated distal tumor was seeded with 10-fold fewer cells compared to the directly treated tumor, resulting in smaller untreated distal tumors compared to the directly treated tumor, representing a decreased challenge for response to immunotherapy, as larger tumors are understood to carry a greater degree of immunosuppressive characteristics (24). In the second study, both directly treated and untreated distal tumors were seeded with identical inoculums of tumor cells, resulting in tumors of similar volume. In both studies, as expected, IT mPH-762 produced a statistically significant inhibition of tumor growth of the directly treated tumor. In both studies, IT mPH-762 treatment also inhibited mean tumor growth of the untreated distal tumor, an abscopal effect that was not observed under treatment with IT NTC (**Figure 3**). The smaller untreated tumors assessed in the first study showed a greater degree of abscopal response to IT mPH-762 compared to the larger tumors in the second study.

These observations indicate that locally administered mouse-targeted PH-762 can stimulate systemic abscopal antitumor efficacy toward untreated distal tumors.

### Intratumoral mPH-762 on-target PD-1 silencing was confined to the directly treated TME suggesting secondary mechanisms of abscopal efficacy toward the untreated distal tumor

To gain insight into mechanisms of action underlying the observed abscopal efficacy of IT mPH-762 toward untreated distal tumors, comparative immunophenotypic characterization was performed on mPH-762-directly treated vs untreated distal TME isolated from the bilateral Hepa1-6 model on Day 14 without leukocyte enrichment (flow gating shown in **Supplementary Figure S8**).

**Figure 4:**
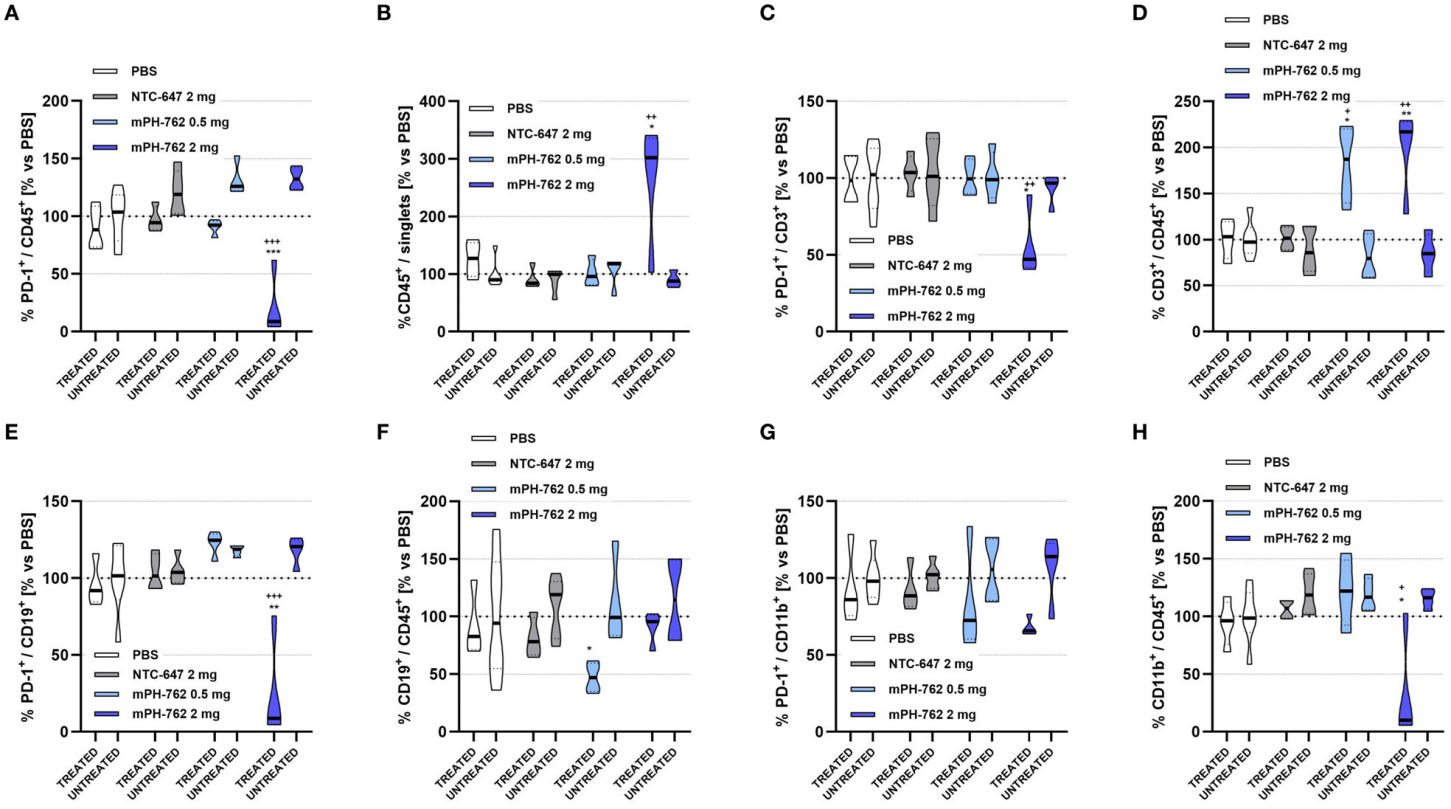
Characterization of on-target immunomodulatory effects of intratumoral mPH-762 in the directly treated vs untreated distal tumor microenvironment isolated on *in vivo* study Day 14 by immunostaining/flow cytometry. Mice were implanted subcutaneously to bilateral flanks with 1e07 Hepa1-6 cells per flank, and treated IT (directly treated tumor only) with mPH-762, NTC or PBS on Days 1, 4, 7, 10 and 13. Violin plots are shown capturing data from each animal with medians indicated by treatment group for directly treated (DT) vs untreated distal tumors (UT). **(A)** Relative percentage (%) of PD-1^+^ / CD45^+^ tumor leukocytes in DT or UT each normalized to those of PBS treated animals (vs PBS). **(B)** Relative overall %CD45^+^ / singlet events in DT or UT vs PBS. **(C)** Relative %PD-1^+^ / CD3+ tumor T cells in DT or UT vs PBS. **(D)** Relative overall %CD3^+^ / CD45^+^ in DT or UT vs PBS. **(E)** Relative %PD-1^+^ / CD19^+^ tumor B cells in DT or UT vs PBS. **(F)** Relative overall %CD19^+^ / CD45^+^ in DT or UT vs PBS. **(G)** Relative %PD-1^+^ / CD11b^+^ tumor myeloid cells in DT or UT vs PBS. **(H)** Relative overall %CD11b^+^ / CD45^+^ in DT or UT vs PBS. Statistical significance of differences in mean AUC were assessed by one-way ANOVA and Tukey’s multiple comparisons *post-hoc* tests. ***p<0.001, **p<0.01, *p<0.05; + = vs NTC; * = vs PBS.

IT mPH-762 again elicited dose-associated on-target reduction of PD-1 surface protein across multiple leukocyte populations in the directly treated (DT) TME, including CD45^+^ TILs overall, CD3^+^ T cells, and CD19^+^ B cells, compared to treatment with IT PBS or NTC. Associated changes in infiltrate levels in the DT TME were often observed: specifically, PD-1 silencing again elicited increased %CD45^+^ TILs, %CD3^+^ T cells. New observations found that the high dose of IT mPH-762 additionally decreased tumor %CD11b^+^ cells, a marker myeloid populations, which are primarily immunosuppressive in the TME (25). In contrast, no evidence of PD-1 silencing was observed for any TIL subpopulation in the untreated distal tumor (**Figure 4**). During our preliminary *in vitro* testing phase, multiple NTC INTASYL constructs were evaluated, and the NTC used in this study was selected based on its consistent minimal impact observed on the parameters under investigation related to PD-1 silencing and TME infiltrate levels. While some slight effects were noted with the chosen NTC, these were substantially lower compared to the robust effects observed with mPH-762.

The lack of on-target PD-1 silencing observed in the untreated distal tumors where antitumor effects were elicited by IT mPH-762 treatment to the contralateral tumor suggests that IT mPH-762 -mediated abscopal efficacy is likely conveyed indirectly via a secondary mechanism of action.

### Intratumoral mPH-762 generates systemic treated-tumor reactive CD8^+^ memory T cells in peripheral lymphoid organs

**Figure 5:**
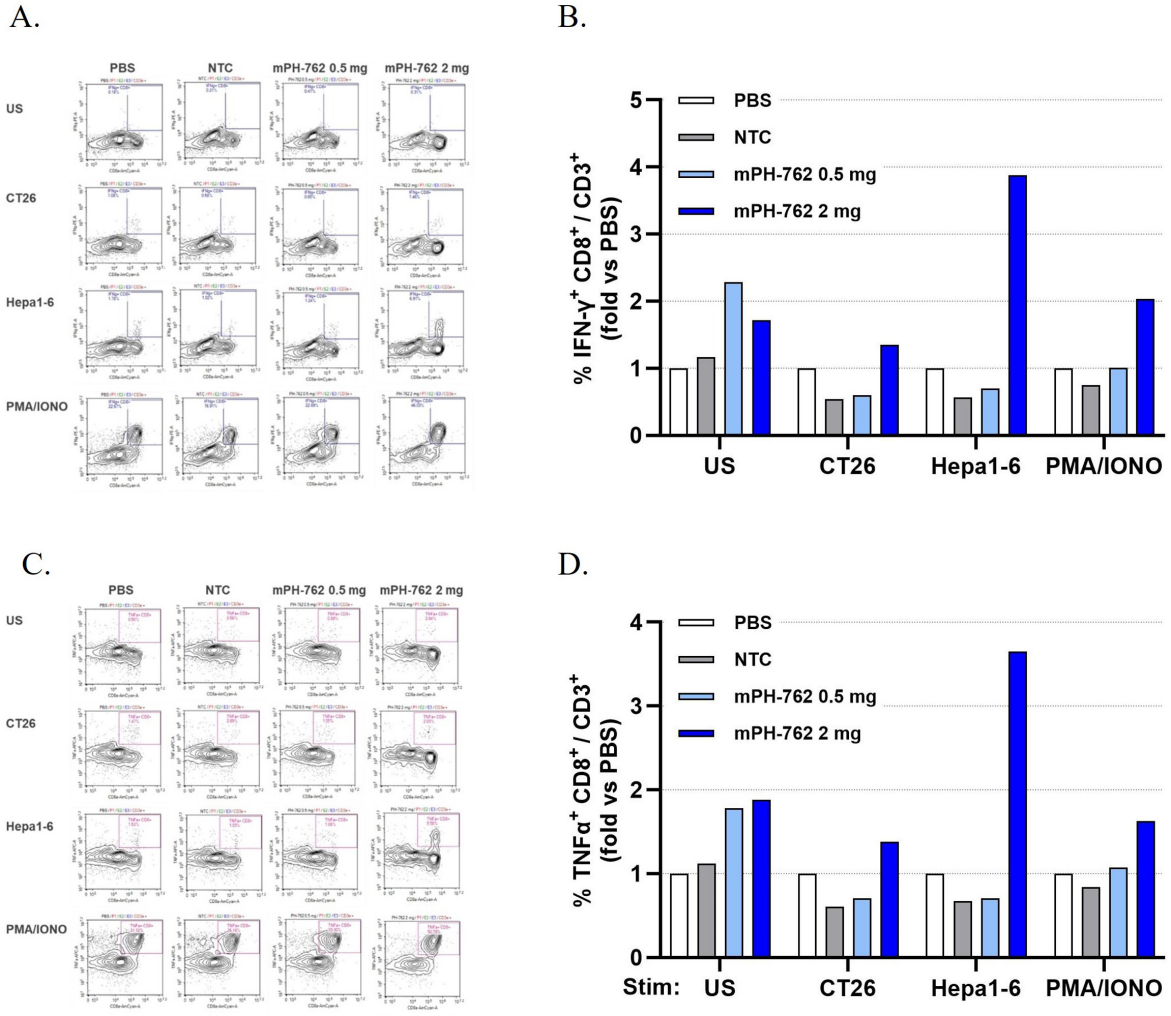
Intratumoral mPH-762 generates systemic treated-tumor reactive CD8+ memory T cells in peripheral lymphoid organs. IT mPH-762 2 mg generates tumor specific systemic CD8^+^ T cells expanded ex vivo from peripheral lymphoid organs (pooled mesenteric lymph nodes and spleens) isolated on Day 14 of the *in vivo* study. Samples from individual mice were pooled by *in vivo* treatment for analysis. **(A)** Flow cytometry plots show memory reactivity of the ex vivo CD8^+^ CD3^+^ T cells isolated from each *in vivo* treatment group as marked by intracellular IFN-γ production in response to matched (Hepa1-6) or mismatched (CT26) tumor cell challenge, or to positive control stimulation (PMA/ionomycin) or unstimulated control conditions. **(B)** Fold-change %IFN-γ ^+^ CD8^+^/CD3^+^ events compared to those observed in the IT PBS treated group by challenge condition. **(C)** Flow cytometry plots show memory reactivity of the ex vivo CD8^+^ CD3^+^ T cells isolated from each *in vivo* treatment group as marked by intracellular TNF-α production of the ex vivo CD8^+^ CD3^+^ T cells in response to each challenge. **(D)** Fold-change of %TNF-α^+^ CD8^+^/CD3^+^ events compared to those observed in the IT PBS treated group by challenge condition.

The ability of local IT mPH-762 to generate systemic memory T cell mediators of anti-tumor immunity was assessed by expanding CD8^+^ T cells isolated on Day 14 from non-tumor peripheral lymphoid organs (PLOs; pooled mesenteric lymph nodes and spleens) isolated from the second (equal sized tumors) bilateral Hepa1-6 tumor study in the presence of Hepa1-6 tumor antigens (irradiated Hepa1-6 cells). Following a 22 days ex vivo expansion, the tumor-specific memory reactivity of PLO CD8^+^ T cells of mice treated IT with mPH-762 was compared to treatment with IT NTC or IT PBS, by challenge with intact matched (Hepa1-6) or mismatched (CT26) tumor cells. Intracellular IFN-γ and TNF-α levels were assayed by immunostaining/flow cytometry as biomarkers of antigen specific CD8^+^ T cell functional memory response (26). Unchallenged or PMA/ionomycin stimulation conditions were included as controls. Only IT mPH-762 treatment at 2 mg generated treated tumor-specifically reactive memory CD8^+^ T cells, as indicated by their increased IFN-γ and TNF-α production specifically in response to Hepa1-6-challenge (but not to an unrelated murine tumor, CT26 challenge); in contrast, similar reactivity was not observed for animals treated IT with PBS, NTC or with mPH-762 at 0.5 mg (**Figure 5**).

These data indicate that IT mPH-762 treatment provided at 2 mg/dose q3d induced systemic tumor specific memory T cells in peripheral lymphoid organs by Day 14 via an on-target mediated effect, suggesting an underlying mechanism of abscopal response elicited by mPH-762 toward untreated distal tumors.

## Discussion

Neoadjuvant ICI with therapeutic antibodies such as those targeting PD-1 has shown promise toward mitigating recurrence and improving outcomes in advanced resectable melanoma, mismatch-repair deficient colon cancer and other cancer indications (10, 11). Neoadjuvant immunotherapy is an attractive treatment paradigm as it has the potential to prime antitumor immunity in the presence of malignant tumors and thereby improve patient outcomes (12). However, systemic ICI mediated irSAEs may represent a new clinical challenge in the neoadjuvant setting, posing a risk for delay of surgery with curative intent.

Here we demonstrate preclinical proof-of-concept for PH-762, a novel self-delivering RNAi therapeutic designed to specifically silence the human PD-1 gene. These studies serve to support the evaluation of PH-762 in clinical application as local immunotherapy for cancer, with the goal of stimulating both local and systemic antitumor immunity, while mitigating the irSAEs conveyed by systemic ICI (ClinicalTrials.gov #NCT06014086). The self-delivering properties of PH-762 make it ideal for application as local immunotherapy.

PH-762 incorporates stabilizing modifications, cholesterol, and a single-stranded phosphorothioate tail, enabling self-delivery via endocytosis. These modifications allow for rapid and efficient uptake by T cells without the need for specialized formulations or delivery vehicles. PH-762 silences its target through the endogenous siRNA pathway, resulting in the sequence-specific degradation of PD-1 mRNA within the RISC and carries negligible risk of sequence mediated off-target effects.

PH-762 mediated functional effects associated with enhanced immune response were observed *in vitro* and *in vivo* (with mPH-762). When administered intratumorally to *in vivo* syngeneic mouse tumor models of injectable indications that may be clinically amenable to surgical resection (melanoma, hepatocellular carcinoma, colon carcinoma), mPH-762 treatment provided enhanced tumor control correlating with dose; degree of response correlated with overall known response of each model to systemic PD-1 inhibition. Functionally, PH-762 was found to increase secretion of IFN-γ, a master activator of the immune response, 4-fold in activated human pan T cells. Ex vivo analyses of the treated TME found that mPH-762 provided dose-associated on-target significant reduction of PD-1 surface protein on tumor CD45^+^ TILs, including CD8^+^ and CD4^+^ T cells. Importantly, IT mPH-762 also increased the overall percentage of CD45^+^ TILs, CD8^+^ and CD4^+^ T cells in the tumor. *In vitro* PH-762 treatment increased secretion of CXCL10, an attractant chemokine for leukocytes, by activated human pan T cells. CXCL10 is a chemokine that serves both as a chemoattractant of T cells and NK cells which express its receptor CXCR3 and a potentiator of T cell cytotoxicity when induced by activation of T cells (27, 28). CXCL10 has been identified as a positive prognostic marker of response to PD-1 immunotherapy in metastatic melanoma (29).

Additionally, mPH-762 treatment decreased PD-1 surface protein in tumor CD19^+^ B cells. Importantly, mPH-762 significantly decreased tumor CD11b^+^ myeloid cells, a primarily immunosuppressive population in the TME (30).

When administered intratumorally, fl-mPH-762 showed biodistribution in the local area of the injected tumor and also throughout the lymphatic system, with lymph nodes proximal to the tumor demonstrating the highest levels of fluorescent-tagged compound. The TME and tumor draining lymph nodes are key interfaces for immune recognition of tumor antigens necessary catalyze anti-tumor immunity.

In this regard, IT mPH-762 was observed to stimulate abscopal antitumor toward untreated distal tumors; perhaps not unexpectedly, smaller untreated distal tumors showed a greater degree of abscopal response compared to larger untreated distal tumors. We performed ex vivo investigations of both the directly treated and untreated TME and found evidence of on-target PD-1 surface protein reduction only in the directly treated TME, suggesting that the direct effects of mPH-762 were largely confined to the directly treated tumor. We hypothesized that the abscopal effects of mPH-762 might be generated locally but conveyed systemically via the immune system. Therefore, we looked for evidence of tumor specific memory immunity in peripheral lymphoid organs (PLOs; pooled tumor draining lymph nodes (tdLNs) and spleens). We found that high dose IT mPH-762 generated tumor-specific memory CD8^+^ T cells in PLOs: these cells showed tumor-specific memory reactivity as marked by expression of intracellular IFN-γ and TNF-α induced by challenge with the same tumor cell line as had been treated IT *in vivo* (Hepa1-6). Specificity was confirmed by lack of reactivity in response challenge with a tumor cell line (CT26) that was not in the *in vivo* study. Expression of IFN-γ and TNF-α was not observed for T cells from animals treated IT with PBS, NTC or with low dose mPH-762. Taken together, these observations suggest a potential mechanism for the abscopal antitumor responses being generated by local treatment with mPH-762 priming an antitumor immune response in lymph nodes.

The goal of immunotherapy for cancer is the generation of long-term tumor-specific immunity (31). This goal has been realized for many patients treated via systemic administration of immune checkpoint targeting antibodies. However, not all patients will respond, and these systemic therapies also elicit risk of irSAEs that may require pause or discontinuation of treatment that may include, if administered in the neoadjuvant setting, risk for delay of timely surgical removal of the tumor.

Intratumoral application of RNAi represents an approach to improve patient outcomes by stimulating antitumor immunity while mitigating irSAEs elicited by systemic immunotherapy. PH-762 is currently being evaluated in a phase 1b clinical study in neoadjuvant treatment of cutaneous malignancies (ClinicalTrials.gov#NCT06014086).

Recent negative clinical trials with modalities such oncolytic virus and TLR-agonists have precipitated a general disappointment with IT immunotherapy presently, where novel immunotherapies did not seem to be as effective as anti-PD1, or did not demonstrate an additional benefit when used with systemic anti-PD1 antibody therapy (32). This suggests that blocking PD-1 at the key tumor:immune interfaces (TME and tdLN) may be critical, however, the properties of antibodies render them ill-suited for application as local immunotherapeutics. The preclinical studies described here along with ongoing clinical studies of intratumoral PH-762 represent initial proof-of-concept of this novel approach to PD-1 inhibition supporting the clinical application of PH-762 as local immunotherapy.

Self-delivering INTASYL compounds such as PH-762 represent a strategy to provide safer and more effective immunotherapy, by focusing immunostimulatory activity to the key sites of tumor:immune interface, the TME and the tdLNs, with the goal of generating durable tumor-specific protective immunity. The safety of INTASYL technology has been previously demonstrated in the clinic (ClinicalTrials.gov NCT0224646 and NCT02599064).

In addition to its precision targeting, self-delivering properties, high stability, and other advantageous properties, INTASYL technology holds great potential as an effective anticancer therapy because multiple key immunosuppressive genes can be targeted at the same time by simple coformulation of multiple INTASYL compounds (33). Indeed, it is well established clinically that targeting multiple immune checkpoints is the most effective immunotherapy, but clinical application is hampered by the emergent systemic toxicities of currently available checkpoint inhibitors. This is also true in the neoadjuvant setting, where combination immunotherapy is the most effective regimen (34) Further, in the adjuvant setting, where benefit/risk ratio is a priority, it is important to have an effective and safe therapy. INTASYL compounds hold the promise of silencing several immune checkpoints or other immune suppressors and/or key tumor drivers in a localized manner to ignite systemic immunity. INTASYL is well suited for adaptable application as monotherapy, in multitargeting coformulations, and/or in combination with current standard of care therapies; such adaptability could enable safer and more individualized options for the clinician, a very attractive concept.

Ultimately, we envision a future where a library of clinically validated INTASYL compounds and combinations would be readily available to patients, such that individualized combination formulations could be easily and rapidly prepared based upon specific biomarkers derived from a biopsy and/or blood sample. In this way, INTASYL has the potential to represent a new approach to precision cancer therapy that is both completely personalized and “off-the-shelf”.

Additionally, INTASYL compounds are relatively inexpensive to produce, are highly stable with a long shelf-life requiring only refrigeration, and can be administered in an office setting, such that patients would no longer need to travel to an infusion center for treatment - all factors expected to substantially increase public accessibility to immunotherapy.

A clinical study (ClinicalTrials.gov#NCT06014086 is currently underway to assess the safety of PH-762 as neoadjuvant therapy for high risk cutaneous malignancies including cutaneous squamous cell carcinoma (cSCC), melanoma and Merkel cell carcinoma where risk of recurrence following surgical removal is high, and there is no approved therapeutic at time of writing.

## Methods

### In silico PDCD1 ortholog mRNA target homology

PH-762 target sequence homology for murine PDCD1 ortholog was performed in silico using the Standard Nucleotide BLAST search tool (“BLASTn” - https://blast.ncbi.nlm.nih.gov/Blast.cgi) using the PH-762 guide strand as the query sequence, using murine reference RNA sequence (refseq_rna; NM_008798.2). PH-762 showed no matching target homology for murine *Pdcd1* and was therefore predicted to carry no target sequence mediated interaction with murine *Pdcd1* mRNA.

### Cells and culture

Cryopreserved human pan T cells (StemCell Technologies #70024) were thawed, washed of freezing medium, and rested for ~24 h prior to each experiment in RPMI 1640 (ThermoFisher #11875-093) supplemented with 10% of fetal bovine serum (FBS; Thermo Fisher cat # 10082-147), 100 U recombinant human IL-2 (rhIL-2; Corning #356043), and 1% penicillin/streptomycin (ThermoFisher #15140-122) in a cell culture incubator at 37°C, 5% CO_2_. At time of treatment through 24 h after application of compound, FBS concentration was reduced to <5% for all experiments.

### Internalization kinetics of fluorescent-PH-762 by human pan T cells

Briefly, cryopreserved human pan T cells (StemCell Technologies #70024) were thawed, resuspended, and rested overnight following standard culture conditions (RPMI 1640 + 10% fetal bovine serum + 1% penicillin/streptomycin + 30 IU/mL recombinant human IL-2, incubations at 37°C; 5% CO_2_.). Following the rest period, cells were activated for 72 h with CD3/CD28-coated activator beads (ThermoFisher #11161D) at a 1:1 bead:cell ratio. After the activation period, the cells were treated with 0.5 µM fl-PH-762 or “fl-canonical RNAi”, a fluorescently labelled, canonical siRNA compound without the structural and chemical changes providing the self-delivering features to the PH-762 compound. Treated cells were cultured for 5 min, 6 h or 24 h and the percentage of fl-PH-762 cellular uptake was quantified using flow cytometry. Also included as a control in this study were unstained cells. All conditions were performed in triplicate.

### PH-762 silencing of PD-1 mRNA by RT-qPCR and surface protein by flow cytometry in activated human pan T cells *in vitro*

Briefly, human pan T cells were rested overnight in culture media in a cell culture incubator at 37°C, 5% CO_2._ The following day, the cells were enumerated and activated with CD3/CD28 coated activator beads at a 1:1 bead: cell ratio and incubated for 72 h. Cells were then treated with either PH-762 or NTC at concentration range of 0- 2 µM and incubated for 72 h. Following the 72 h incubation, the cells were pelleted and resuspended in fresh culture media that did not contain PH-762. The media was refreshed every 72 h. mRNA was isolated by RNeasy Mini Kit (Qiagen #74106) at 3 days (72 h), 6 days and 9 days post treatment and yield concentrations determined by Nanodrop™ spectrophotometer. Input was normalized and PDCD1 mRNA levels were detected relative to reference gene PPIB by TaqMan™ RNA-to-CT™ 1-Step Kit (Thermo Fisher #4392938) and human PDCD1 TaqMan assay (ThermoFisher #4331182 Hs01550088_m1, FAM-MGB) and human PPIB TaqMan assay (ThermoFisher #4331182 Hs00168719_m1, FAM-MGB) on a QuantStudio3 RT-qPCR instrument (Applied Biosystems).

PD-1 protein expression was assessed by flow cytometry on a NovoCyte flow cytometer (Agilent) and analyzed using NovoExpress software (Agilent) following immunostaining with APC anti-human CD279 (PD-1) antibody (Biolegend # 367406) at 72 h, 7 days, and 10 days. Experiments were conducted using cells from three independent donors in triplicate.

### mPH-762 silencing of murine PD-1 mRNA in EL4 murine T cells *in vitro*

Briefly, cryopreserved EL4 cells (ATCC #TIB-39) were thawed according to ATCC protocol into standard culture media (Dulbecco’s Modified Eagle’s Medium + 10% FBS + 1% Pen/Strep) and rested overnight in a cell culture incubator at 37°C, 5% CO_2._ resuspended at a density of 0.4x10^6^ cells/mL in transfection media (Dulbecco’s Modified Eagle’s Medium + 5% FBS + 1% Pen/Strep). Appropriate volumes of mPH-762 were added to each well of a 24-well tissue culture plate. EL4 cells were added to the plate containing the INTASYL compounds for final concentration of 2 µM, 1 µM, and 0.5 µM. Plates were rocked gently to ensure thorough mixing of cells and compound. Transfections were performed in triplicate for each condition. Cells were kept at 37°C and 5% CO_2_ for the duration of the experiment. After 72 hours, cells were collected for analysis PD-1 mRNA expression by RT-qPCR.

mRNA was isolated by RNeasy Mini Kit (Qiagen #74106) at 3 days (72 h), 6 days and 9 days post treatment and yield concentrations determined by Nanodrop™ spectrophotometer. 50-100 ng input was used to detect Pdcd1 mRNA levels relative to reference gene Tbp by TaqMan™ RNA-to-CT™ 1-Step Kit (Thermo Fisher #4392938) and mouse Pdcd1 TaqMan assay (ThermoFisher #4331182 Mm01285677_g1, FAM-MGB) and mouse Tbp TaqMan assay (ThermoFisher #4331182 Mm01277042_m1, FAM-MGB) on a QuantStudio3 RT-qPCR instrument (Applied Biosystems).

### Functional cytokine release via ELISA or cytometric bead array assay

Human pan T cells were stimulated with plate-bound murine anti-CD3 (OKT3) at 100 ng/mL and simultaneously treated with 2 µM PH-762 or 2 µM NTC. Unstimulated, PBS-treated cells (US) and OKT3 stimulated PBS-treated (UTC) conditions were included as controls. Secreted IFN-γ was detected on Day 3 post-stimulation/treatment by human IFN-γ ELISA (R&D Systems #DIF50C). Secreted CXCL10 was detected on Day 3 post-stimulation/treatment by cytometric bead array (CBA) assay (Biolegend #740930) according to manufacturer’s instructions.

To ensure that differences in secreted cytokine levels were not a result of differences in viable cell numbers across the various conditions, a 7-aminoactinomycin D (7AAD) cell viability assay was performed in triplicate for each condition for pan T cells derived from each donor. Briefly, cells were stained 1:50 with 7AAD in the dark and 7AAD^+/-^ cells were assessed by flow cytometry without washing. Cells stimulated with OKT and treated with PBS (UTC) or NTC showed a slight drop in overall cell viability to ~80% of US cells; cells treated with PH-762 showed cell viability that was further reduced to ~60% of US. Although differences in cell viability did not reach statistical significance, ELISA and CBA assay readouts of IFN-γ and CXCL10 were normalized across conditions to reflect secreted cytokine production from an equivalent number of viable cells.

PD-1 silencing was confirmed at the mRNA level by RT-qPCR as described above on Day 3 and at the protein level by flow cytometry at Day 7 as described above.

### Syngeneic tumor models

All animal studies were performed at Pharma Models LLC (Marlborough, MA) under validated protocols in accordance with their IACUC. For all studies, mice were humanly euthanized by CO_2_ chamber when tumors reached predetermined threshold volume. For all studies, tumor volumes were calculated from length and width measurements using the standard ellipsoid equation V = (Length x Width^2^)/2.

A PD-1-inhibition responsive syngeneic subcutaneous (SC) Hepa1-6 model of murine hepatocellular carcinoma was performed in female C57BL/6 mice. Animals (n = 12/group) were randomized into treatment groups around a common mean tumor volume of ~150 mm^3^ prior to commencement of treatment on Day 1. Treatments were administered on Days 1, 3, 7, 10 and 14 by IT injection of 0.05 mL mPH-762 (0.5 mg or 2 mg), NTC (2 mg) or PBS vehicle, or by intraperitoneal (IP) injection for anti-PD-1 monoclonal antibody (mAb) (IP; 200 µg/dose) as a systemic positive control. Animals were weighed and observed daily, and tumors measured 3x/week. A satellite group (n = 6/group) was euthanized on Day 13 and tumors isolated and processed for flow cytometry based immunophenotypic characterization of the tumor microenvironment (TME).

A PD-1-inhibition moderately-responsive syngeneic SC B16-ovalbumin (B16-OVA) model of murine melanoma was performed in female C57BL/6 mice. Animals (n = 6/group) were randomized into treatment groups around a common mean tumor volume of ~150 mm^3^ prior to commencement of treatment on Day 1. Treatments were administered on Days 1, 4, and 7 by IT injection of 0.05 mL mPH-762 (0.1 mg, 0.25 mg, 0.5 mg, 1 mg or 2 mg) or PBS vehicle. Animals were weighed and observed daily, and tumors measured 3x/week.

A PD-1-inhibition modestly-responsive syngeneic SC CT26 model of murine colon carcinoma was performed in BALB/c mice. Animals (n = 8/group) were randomized into treatment groups around a common mean tumor volume of ~150 mm^3^ prior to commencement of treatment on Day 1. Treatments were administered on Days 1, 3, 7, 10 by IT injection of 0.05 mL mPH-762 or by systemic intraperitoneal (IP) injection anti-PD-1 antibody as a positive control. Group 1 received PBS vehicle. Group 2 received mPH-762 (1 mg/dose). Group 3 received anti-PD-1 mAb (IP; 200 µg/dose). Animals were weighed daily, tumors were measured 3x/week.

A bilateral syngeneic PD-1 inhibitor-responsive Hepa1-6 model of murine hepatoma was performed in female C57BL/6J mice. Animals (n = 5/group) were implanted with 1e07 Hepa1-6 cells on both left (treated) and right (untreated) flanks. Animals were randomized into treatment groups around a common mean left (treated) tumor volume of ~150 mm^3^ prior to commencement of treatment on Day 1. Treatments were administered to the left (treated) tumor only, on Days 1, 4, 7, 10, and 13 (Q3D x5) by IT injection of 0.05 mL mPH-762 at 0.5 mg (10 mg/mL) and 2 mg (40 mg/mL) or with vehicle (PBS) as negative control. Animals were weighed daily, and both left (treated) and right (untreated) flank tumors were measured 3x/week. Both directly treated and untreated distal tumors were isolated on Day 14 and processed for flow cytometry based immunophenotypic characterization of the directly treated or untreated distal TME. Additionally on Day 14, mesenteric lymph nodes and spleens were isolated as pooled samples per mouse and dissociated to single cell suspension as described above to enable expansion of systemic tumor specific T cells from peripheral lymphoid organs.

### Ex vivo immunophenotypic characterization of tumor microenvironment

Tumors isolated from *in vivo* syngeneic models as indicated were dissociated in small batches in 10 mL (15 mL conical) pre-warmed dissociation solution (RPMI medium, Collagenase (0.7 mg/mL), and DNase I (30 U/mL)) for 45 min @ 37°C on a rotator and homogenized to single-cell suspension with a 14G sterile pipetting needle attached to a 30 mL syringe and passed through a 70 μm cell strainer before the digestion was stopped by addition of 2 mL of FBS. Cells were pelleted by centrifugation and resuspended in 5 mL of 1X red blood cell (RBC) Lysis Buffer and incubated for 3-4 minutes at room temperature (RT). The cells were then passed through a 40 µm cell strainer and pelleted by centrifugation, resuspended in 5 mL Isolation Media (RPMI + 10% fetal bovine serum (FBS) + 1X penicillin/streptomycin antibiotics) and counted by flow cytometer. The cell suspension was then slowly and carefully layered onto 5 mL Lympholyte^®^-M (Cedarlane Cat#CL5030) in a 15 mL conical to produce a two-phase gradient and centrifuged for 20 min at 1500 xG at Room Temperature with natural (no breaking) deceleration at spin end. The lymphocyte-enriched middle layer (~2 mL) and tumor cell-enriched lower layer were collected into separate tubes and the Lymopholyte^®^-M diluted away with 8 mL RPMI. Cells were pelleted as above, the supernatant removed and cells were resuspended in PBS for enumeration by flow cytometer (Novocyte; ACEA Biosciences). Cells were plated into 96-well plate at 1e06 cells/mL in Cell Staining Buffer (Biolegend cat # 420201) for immunophenotyping with antibody panels that included: anti-mouse CD45-BV421 (Biolegend cat # 103134) or anti-mouse CD45.2-Pacific Blue™ (Biolegend cat # 109820); anti-mouse CD3-PE/Cy5.5 (Biolegend cat # 100218) or anti-mouse CD3-BV510 (Biolegend cat # 300448), anti-mouse CD4-APC/Cy7 (Biolegend cat # 100414), anti-mouse CD8-AF700 (Biolegend cat # 100730), anti-mouse CD19-APC-Cy7 (Biolegend cat # 115530), anti-mouse NK1.1-APC (Biolegend cat # 108710), anti-mouse CD11b-PE-Cy7, anti-mouse PD-1-FITC (Biolegend cat # 135214) or anti-human/mouse PD-1-AF488 (R&D Systems cat # FAB7738N) and incubated for 60 minutes in the dark at room temperature. Post-incubation, cells were washed twice with PBS. The samples were resuspended in 125 μL staining buffer for acquisition on the flow cytometer. Spectral compensation was enabled by single staining UltraComp eBeads™ (ThermoFisher #01-2222-41) with each antibody. An unstained control sample (no antibody) served for gating purposes. Lymphoid cell populations of interest were enriched based on forward versus side scatter. The flow cytometer collected events by running a fixed volume of 100 μl of each sample. Flow cytometric data acquisition was performed by Novocyte flow cytometer (Agilent) and analyzed with NovoExpress™ software (version 1.3.0, Agilent).

### Ex vivo expansion of tumor specific T cells and memory reactivity challenge

Mesenteric lymph nodes (mLN) and spleens (SP) isolated on Day 14 from mice treated in the bilateral Hepa1-6 *in vivo* study were dissociated by GentleMACS™ dissociator and passed through a 70 µm cell strainer to produce a single cell suspension. Cells were pelleted by centrifugation and resuspended in 1-2 mL of 1X red blood cell (RBC) Lysis Buffer and incubated for ~5 minutes at room temperature (RT). Cells in RBC lysis buffer were diluted to 5 mL with Isolation Media (RPMI + 2% FBS + 1% pen/strep), washed 1X with PBS and frozen in 90% FBS/10% DMSO for later use.

One day prior to initiation of expansion, Hepa1-6 cells in culture were trypsinized and resuspended in complete RPMI (ATCC formulation; “ATCC cRPMI”) containing 1% pen/strep and 10% FBS and irradiated (IR) with 100 Gy (IR performed at Pharma Models LLC, by focused X-ray irradiator), enumerated by fixed volume flow cytometer (Novocyte; Agilent) and plated into 24-well plates at a density of 2e05 cells/well. Frozen mLN/SP cells were thawed and rested overnight in ATCC cRPMI Following overnight culture, viable cells (based on forward scatter (FSC) and side scatter (SSC)) were enumerated by Novocyte flow cytometer. 5e06 viable SP/LNs per mouse were plated into individual wells on top of the IR Hepa1-6 cells in the presence of recombinant murine IL-7 (rm-IL-7; 5 ng/mL). On Day 3 low-dose recombinant murine IL-2 (rm-IL-2; 20 U/mL) was added. Half-medium change and supplementation of cytokines was performed every 3 days. After 10 days, T cells were harvested, washed 1X with PBS, and replated in fresh ATCC cRPMI and cytokines as previously onto IR (100 Gy) Hepa1-6 cells that were irradiated and plated one day prior (Day 9). On Day 22 of expansion, T cells were harvested and enumerated by Novocyte flow cytometer. 2.5e04 T cells from each well/mouse were plated into four (4) separate wells of a 96-well flat bottom plate, representing four tumor specific reactivity challenge stimulation conditions: 1) unstimulated control, 2) CT26 BALB/c murine colon cancer cell challenge (mismatched; mice were not previously exposed to this tumor, derived from BALB/c as opposed to C57BL/6 mice); 3) Hepa1-6 cell challenge (matched, tumor-experienced challenge) or 4) PMA/ionomycin (PMA/iono; as Cell Stimulation Cocktail 500X [eBioscience/Thermo Fisher 00-4970-93]) stimulation as +control. For the tumor cell challenges, tumor cells in culture were trypsinized, enumerated by Novocyte flow cytometer, and 2.5e03 viable cells (FSC/SSC) plated into the appropriate wells for a final T cell effector (E) to tumor target (T) cell ratio (E:T) of 10:1 in a total volume of 0.1 mL ATCC cRPMI. PMA/iono was added to the appropriate wells in a final total volume of 0.1 mL 1X final concentration; unstimulated wells were brought to final volume of 0.1 mL with ATCC cRPMI. All samples/conditions were incubated for ~6 h in a tissue culture incubator @37°C, 5% CO_2_. At ~3 h post-initiation of reactivity challenge, GolgiStop (monesin) and GolgiPlug (brefeldin A) were each added in a combined volume of 50 µL for 1 µL/mL final concentration of each. After ~6 h post-initiation of reactivity challenge, immunostaining antibodies (Abs) were added in a total volume of 25 µL such that the total volume/well was 0.2 mL with a final concentration of 1:100 for anti-mouse CD3e-PE-Cy5 (Biolegend cat # 561089) and 1:200 for anti-mouse CD8a-BV510 (Biolegend cat # 100752) and anti-mouse CD4-AF700 (Biolegend cat # 100536) and incubated for and additional ~30 min @37°C, 5% CO_2_. Post-incubation, cells were pelleted @ 350 XG for 5 min washed twice with PBS and fixed with 0.1 mL eBioscience Foxp3/transcription factor fixation/permeabilization (perm) buffer for ~1 h @ 4°C in the dark. Wells were filled to 0.2 mL with perm buffer without fixative, washed two more times with perm buffer and resuspended in 50 µL perm buffer for intracellular (IC) staining using anti-mouse IFN-γ-PE (Biolegend cat # 505808) and anti-mouse TNF-α (Biolegend cat # 506308) diluted in perm buffer to 2X final concentration (1:100 final dilution) and 50 µL/well applied to cells for a total volume of 0.1 mL/well. Cells/IC staining Abs were incubated ~1 h @ 4°C in the dark. Cells were then again pelleted by centrifugation and washed 2X with 200 µL perm buffer. Due to the low number of cells/well, samples were pooled by *in vivo* treatment group per condition in 100 µL perm buffer for flow cytometry analysis by Novocyte flow cytometer with 75 µL/well sampled. Resultant data was analyzed with NovoExpress™ software (version 1.3.0).

### Statistical analyses

Group means ± SEM were intercompared across treatment conditions by one way ANOVA and Tukey’s or Dunnett’s multiple comparisons *post-hoc* tests using Prism 9 (GraphPad). Statistical significance was considered achieved for p < 0.05. (*p<0.05; **p<0.01; ***p<0.001; ****p<0.0001). “*” = vs US; “#” = vs UTC; “+” = vs NTC.

Patients or the public were not involved in the design, or conduct, or reporting, or dissemination plans of our research.

## Data Availability

The raw data supporting the conclusions of this article will be made available by the authors, without undue reservation.
