# Supplementary material for: Self-delivering RNAi immunotherapeutic PH-762 silences PD-1 to generate local and abscopal antitumor efficacy
Source: Front Immunol. 2024 Dec 4;15:1501679. doi: 10.3389/fimmu.2024.1501679 (PMC11652358; doi:10.3389/fimmu.2024.1501679)
Supplement: Supplementary file 1 [file DataSheet1.docx]

**Supplementary Figures**

Figure S1

**A. B.**

Figure S1

PH-762 does not silence GRAMD4 mRNA at nontoxic concentrations in CD3/CD28 bead-activated human pan T cells. **A.** GRAMD4 or **B.** PD-1 (PDCD1) mRNA levels after 72 h of treatment with PH-762 at concentrations of 1-5 µM (RT-qPCR, reference gene PPIB). Percent change relative to PBS vehicle control (Untreated Control) is shown ± SEM (N=6). IC_50_ calculations were calculated using [Inhibitor] vs. normalized response – variable slope performed in GraphPad Prism. Data is combined results of two experiments (N=6).

Figure S2

**A. B. C.**


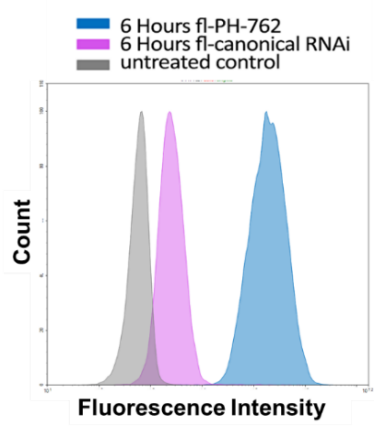


D. E.

F. G.

**Figure S2:** **PH-762 is rapidly and efficiently taken up by human pan T cells and silences PD-1 to enhance functional biomarkers of TCR-mediated activation.**

**A.** Mean fluorescence intensity (MFI) over time of human pan T cells treated with fl-PH-762 quantified by flow cytometry. **B.** The data shown in A. displayed as fold-change at each time point. **C.** Histograms depict count (Y-axis) and fluorescence intensity (X-axis) flow cytometry data at 6 h post-treatment, suggesting positive signal in 100% of cells. Grey = unstained, pink = canonical siRNA, blue = fl-PH-762. **D.** PH-762 silencing of PD-1 mRNA or **E.** protein at 72 h post-treatment in CD3/CD28-bead activated primary human pan T cells, representative data for single donor. NTC is shown as non-targeting control. Percentage of PD-1 mRNA relative to PBS-treated controls (UTC) is shown ± SEM (N = 3) as determined by 2-ΔΔCt RT-qPCR; Percentage of PD-1 protein relative to PBS-treated controls (UTC) is shown ± SEM (N = 3) as determined by flow cytometry. IC50 values were calculated by least squares fit. . PH-762 enhances stimulation-induced IFN-γ and CXCL10 secretion by human pan T cells**.** Secreted **F.** IFN-γ or **G.** CXCL10 in the conditioned media of human pan T cells at 72 h post-treatment with PH-762 or NTC (2 µM) and stimulation with plate-bound OKT3. Viable cell normalized levels are shown by condition ± SEM (N = 3/donor). Symbols distinguish the individual donors. The statistical significance of differences in group means were intercompared by one way ANOVA and Tukey's multiple comparisons *post-hoc* tests. ****p<0.0001, ***p<0.001, **p<0.01, *p<0.05. * = vs US, # = vs UTC, + = vs NTC.

Figure S3

A. B.

C. D.

E.

**Figure S3 A.** IL-2 **B.** IL-6 **C.** IL-10 **D.** TNF-α or **E.** IFN-γ cytokine levels in cell culture supernatants of human PBMCs treated with soluble test articles from each donor in different treatment groups. Each symbol represents the average of the triplicates for each donor. The horizontal bar represents the median value of all donors (10 total) within its respective treatment group.

Figure S4

**Figure S4:** Murine PD-1 mRNA relative levels in murine EL4 T lymphocytes following 72 h of treatment with mPH-762 quantified by 2^-ΔΔCt^ RT-qPCR; fold-change relative to untreated controls (UTC) is shown ± SEM (N = 3). Patterns distinguish 3 independent experiments. Mean relative PD-1 mRNA levels of mPH-762 treated cells were compared to those of untreated cells by two-way ANOVA and Tukey’s multiple comparisons *post-hoc* tests. ****p<0.0001, ***p<0.001, **p<0.01, *p<0.05. Symbols distinguish individual experiments.

Figure S5

A.

B.

C.

### Figure S5: IT mPH-762 treatment is well tolerated in syngeneic tumor models

Body weights were measured daily. Group mean percentage (%) of body weight change (± SEM) over time (left) or cumulative Area under the curve (AUC) (right) following IT mPH-762 treatment. Violin plots with individual animals and medians are shown. mPH-762 was administered IT q3d as indicated by arrows. Anti-PD-1 mAb control was administered IP q3d. **A.** Hepa1-6 model **B.** B16-OVA model. **C.** CT26 model. AUC was calculated for each animal by trapezoidal transformation over the indicated time period. Statistical significance of differences in mean AUC were assessed by one-way ANOVA and Tukey’s multiple comparisons post-hoc tests.

Figure S6 Fluorescent mPH-762 biodistribution following IT administration in mice

**A.**

**
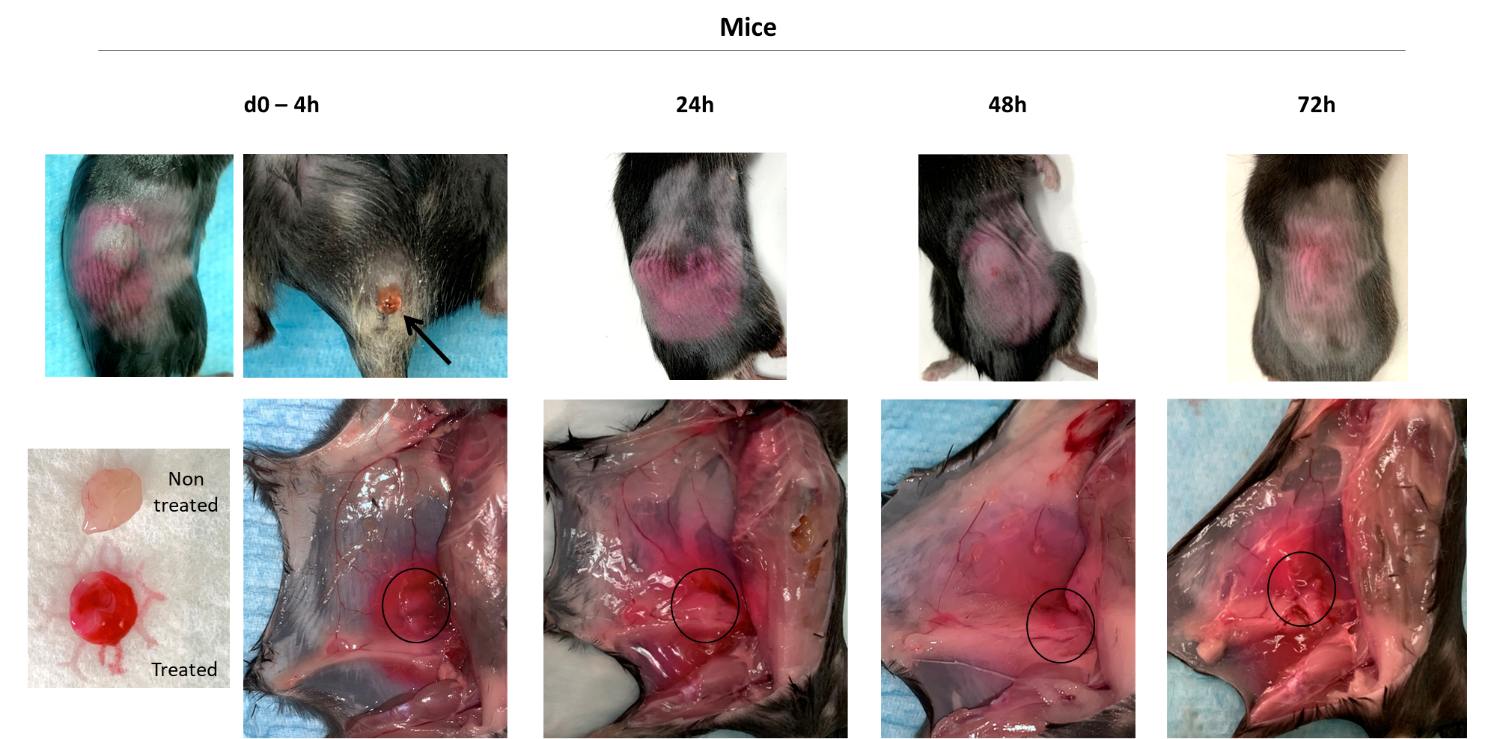
**

**B.**


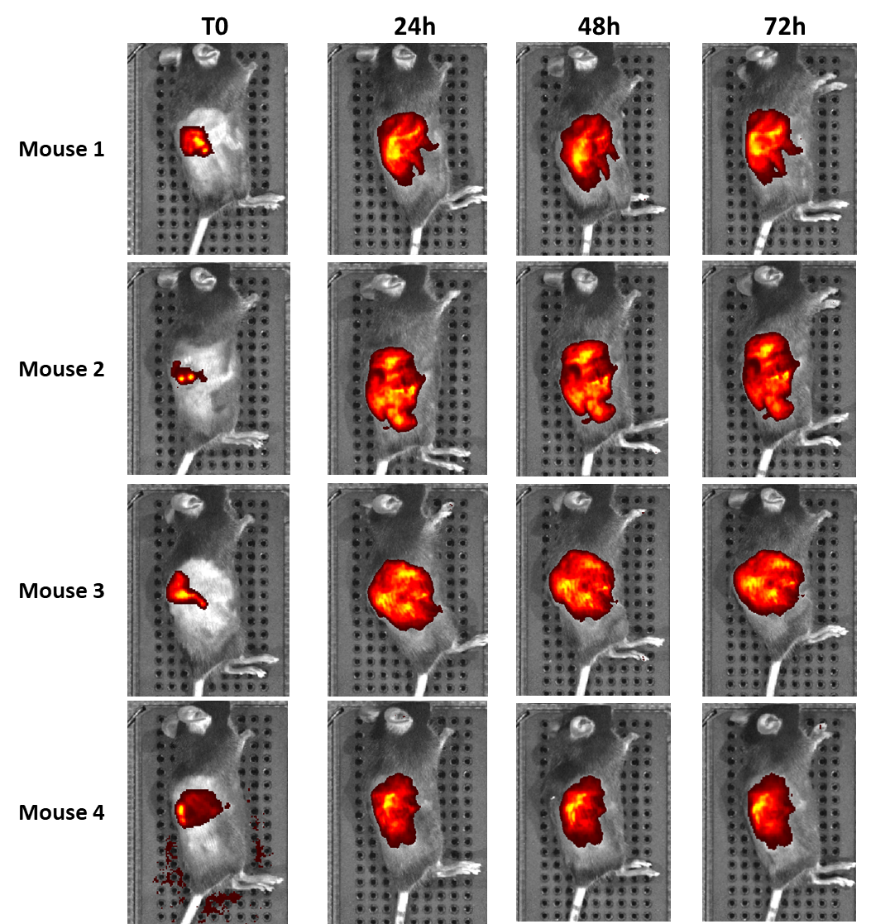


**C.**


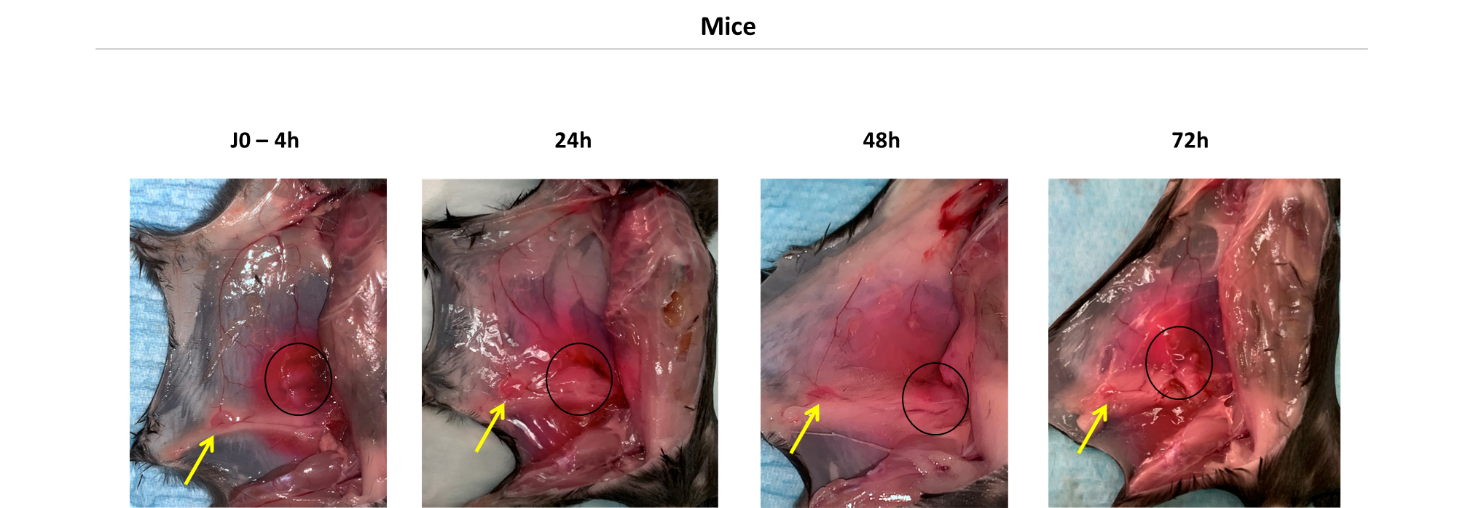


**D.**

**
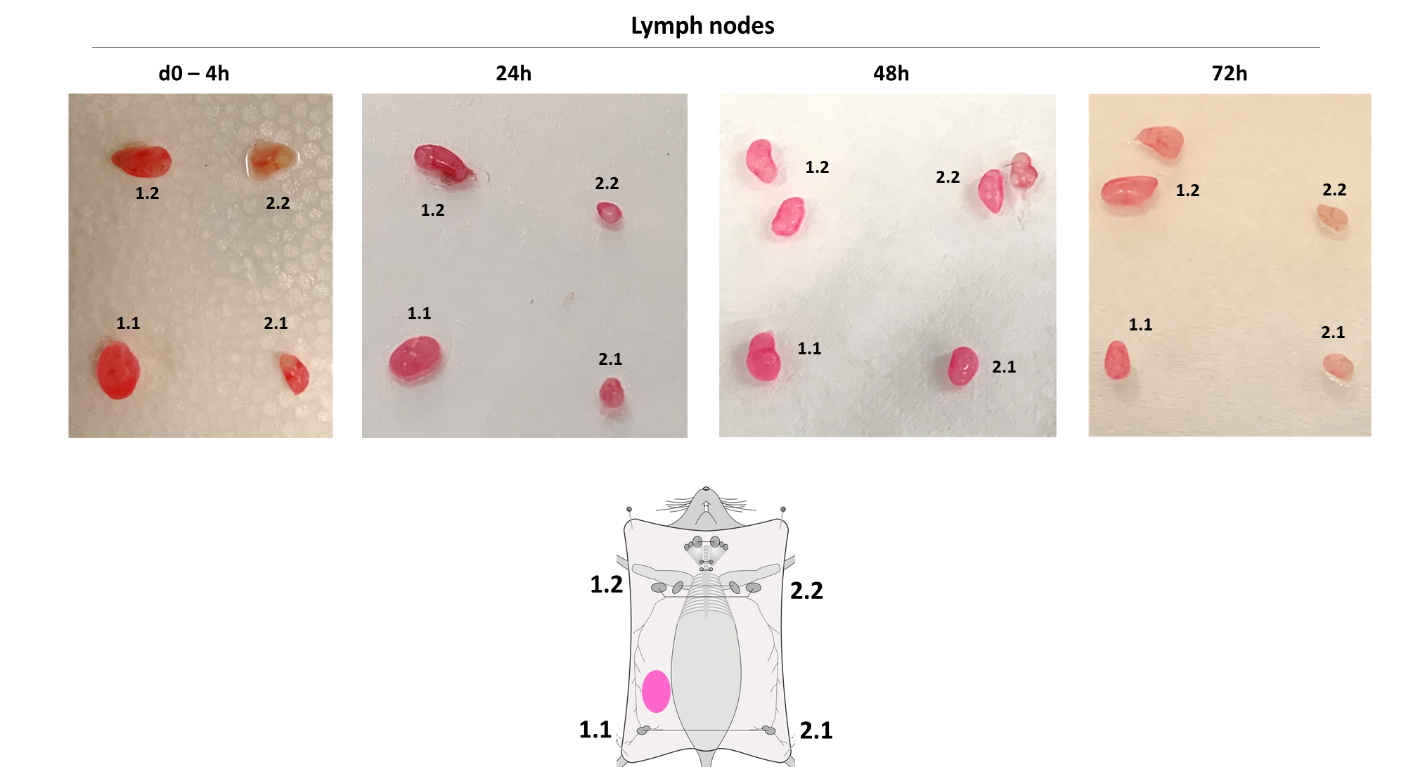
**

Figure S6

Mice were inoculated with BP cells in the right flank and were monitored until the tumors mean volume reached 150 mm3. Fluorescent mPH-762 was then dosed via intra-tumoral (IT) injection of 50 µL/dose for a single dose of 2 mg. Fluorescence was assessed by A. by naked eye as pink coloration of the skin or B. IVIS® SpectrumCT imaging system. Mice were then euthanized and the tumors (C., black circle) and the lymph nodes (C., yellow arrow and D.) were collected. The percentage (%) of fluorescence-positive cells found in lymph nodes of various distance from the tumor was quantified by flow cytometry at 24 h, 48 h and 72 h post administration. E. % fluorescence-positive total cells from selected lymph node over time. F. % fluorescence-positive CD8+ cells from various lymph nodes over time. G. % fluorescence-positive CD4+ cells from various lymph nodes over time.

Figure S7


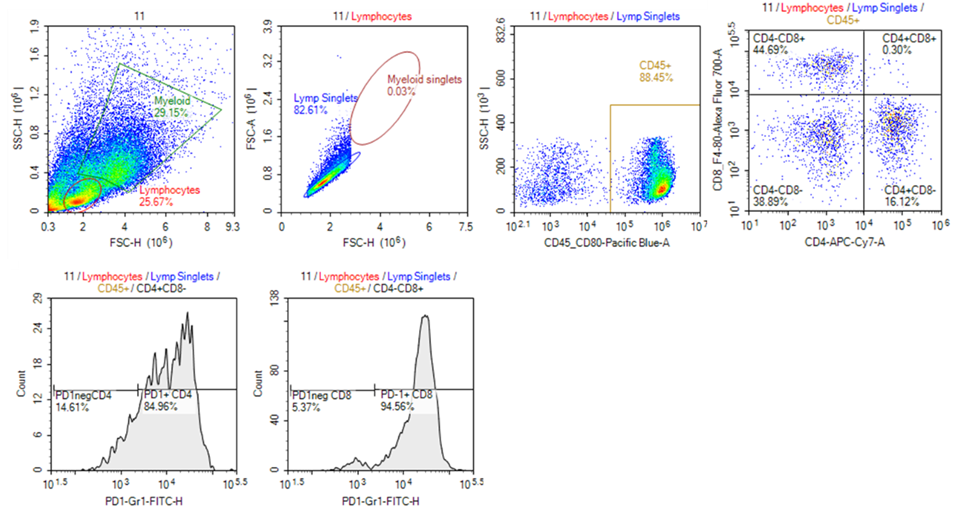


Figure S7

Ex vivo tumor lymphocyte Gating – leukocyte enriched

Figure S8


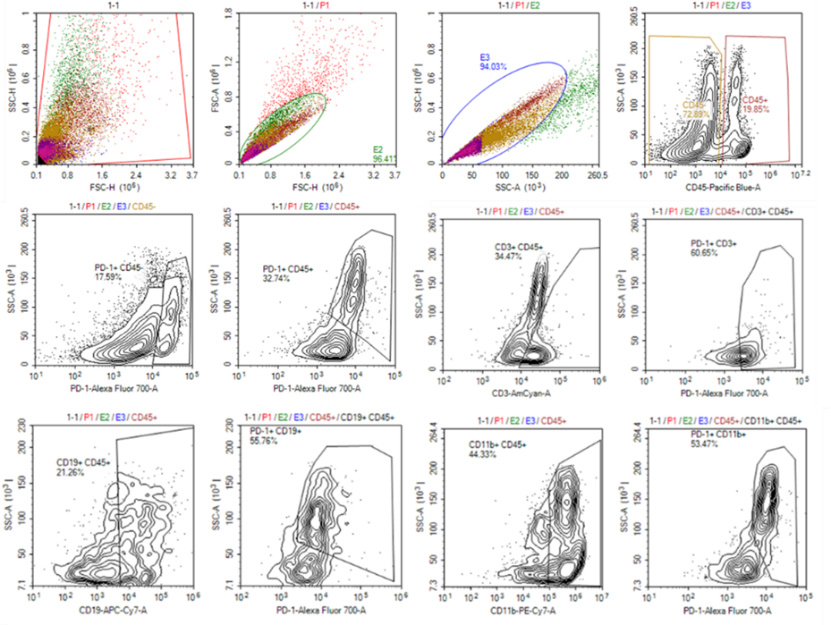


Figure S8

Ex vivo tumor lymphocyte gating – non-leukocyte enriched
